# Folksong based appraisal of bioecocultural heritage of sorghum (*Sorghum bicolor *(L.) Moench): A new approach in ethnobiology

**DOI:** 10.1186/1746-4269-5-19

**Published:** 2009-07-03

**Authors:** Firew Mekbib

**Affiliations:** 1 Haramaya University, PO Box 138, Dire Dawa, Ethiopia; 2 Norwegian University of Life Sciences, Department of Plant and Environmental Sciences, PO Box 5503, N-1432, Aas, Norway; 3 PO Box 485 code 1250, Addis Ababa, Ethiopia

## Abstract

**Background:**

Sorghum is one of the main staple crops for the world's poorest and most food insecure people. As Ethiopia is the centre of origin and diversity for sorghum, the crop has been cultivated for thousands of years and hence the heritage of the crop is expected to be rich. Folksong based appraisal of bioecocultural heritage has not been done before.

**Methods:**

In order to assess the bioecocultural heritage of sorghum by folksongs various research methods were employed. These included focus group discussions with 360 farmers, direct on-farm participatory monitoring and observation with 120 farmers, and key informant interviews with 60 farmers and development agents. Relevant secondary data was also collected from the museum curators and historians.

**Results:**

The crop is intimately associated with the life of the farmers. The association of sorghum with the farmers from seed selection to utilization is presented using folksongs. These include both tune and textual (ballad stories or poems) types. Folksongs described how farmers maintain a number of varieties on-farm for many biological, socio-economic, ecological, ethnological and cultural reasons. Farmers describe sorghum as follows: *Leaf number is less than twenty; Panicle hold a thousand seeds; a clever farmer takes hold of it*. In addition, they described the various farmers' varieties ethnobotanically by songs. The relative importance of sorghum vis-à-vis others crops is similarly explained in folksong terms.

**Conclusion:**

The qualitative description of farmers' characterisation of the crop systems based on folksongs is a new system of appraising farmers' bioecocultural heritage. Hence, researchers, in addition to formal and quantitative descriptions, should use the folksong system for enhanced characterisation and utilization of bioecocultural heritages. In general, the salient characteristics of the folksongs used in describing the bioecocultural heritages are their oral traditions, varied function, communal or individual recreation and message transmissions.

## Background

Sorghum is one of the main staple crops for the world's poorest and most food insecure people. The crop is genetically suited to hot and dry agro-ecologies where it is difficult to grow other food grains. Developing countries account for roughly 90% of the world's sorghum area and 77% of the total output [1]. In the developing countries, much of the crop is grown by small-scale farming households operating at the margins of subsistence.

Ethiopia is the centre of origin and diversity for sorghum [2], The crop has been cultivated for many thousands of years and hence indigenous knowledge-based sorghum classification and naming has rooted traditions. Sorghum ranks fourth in area coverage after tef (*Eragrostis tef *(Zucc.) Trotter), maize (*Zea mays *L.), and wheat (*Triticum spp*.). It is grown on 1,253,620 ha with a total production of 1,715,954 tons [3] and it accounts 14.2% and 13.6% of the crop area and production respectively. As a result, there are over 3,674,865 farmers households dependent on sorghum production.

The mentioning of the name of sorghum dates back to some writings in 1305 A.D. The earliest known reference was by Ruel (1537) as cited in [4]. The current formal taxonomic concept of the sorghum genus and species agrees with the one established by Moench. All the different names given by the various taxonomists are hence taken as synonyms for *S. bicolor *(L.) Moench.

Farmers have been describing sorghum by various folk taxonomic systems since its domestication. This happen in the course of varietal naming, selection, growing, protection, storage and utilization, which spans from the field to the table [5]. Farmers characterise their varieties both qualitatively and quantitatively. However, farmers' qualitative depiction of crop and cropping system is rarely given importance in research. This description is commonly encompassed in the ethnobotanical and sociological description of the crop-farmer-environment interactions. Despite the intimate crop-people-environment association in the centre of diversity and the salient importance of the knowledge of ethnobotanical description, no published information was available for folksong-based description of bioecocultural heritages in sorghum in Ethiopia. Folksongs include both tune and textual (ballad stories or poems) types. Hence, the objective of this study was to assess, characterise and document the folksong-based description of bioecocultural heritages of sorghum diversity, production, management and utilization in the centre of diversity, Ethiopia.

## Research methodology

### Study site selection

**Figure 1:**
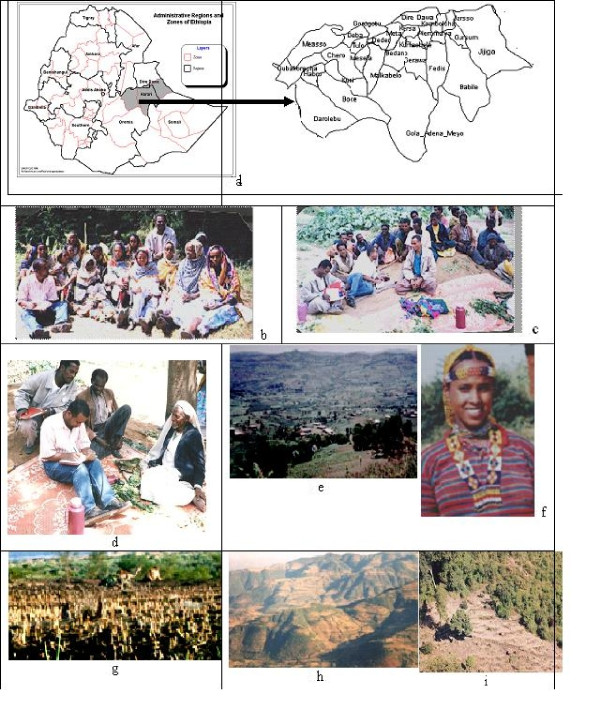
**1a. Map of the study area**. **1b**. Women focus group discussion **1c**. Men focus group discussion. Note the Khat (*Chata edulis *Forskk)-a mild drug crop commonly chewed for its stimulating tender leaves and buds **1d**. Key informant interview with 80 years old farmer. **1e**. Various *Gandas *in *Hirna *area, Ethiopia. Note the *tikul*, group of houses covered with corrugated sheets of iron or grass-sorghum thatching, clustered all over the areas. **1f**. An *Oromo *girl from *Babile wereda *with traditional dress **1g**. Drylowlands of *Goloda*. Note the camel near the harvested sorghum field with stumps. Sorghum. due its drought resistnace, is sometimes called 'the camel crops of cereals' **1h**. Eastern Ethiopia cool highlands topography associated with sorghum. The undulating hills and valleys are partly responsible for genetic differentiation and isolation **1i**. Shifting cultivation into the natural forest area in the highlands. *Zigita *area, West Hararghe, Ethiopia.

Eastern Ethiopia (Figure 1a) was selected for the following reasons:

1. The crop has coexisted with the people for millennia and sorghum production is pre-dominantly based on farmers' varieties. Hence, farmers are expected to have an established folksong-based system of appraisal of sorghum.

2. The region had rich on-farm genetic resources of sorghum that made it suitable to study ethnobotanical descriptions [5] of sorghum.

3. In eastern Ethiopia, there are over 635, 462 households dependent on sorghum production, which helps to document the folksongs of various farmers in different ecological setups.

Diverse research methods were employed in order to understand the many facets of the bioecocultural heritage of sorghum in the centre of diversity.

### Survey methods

Different survey methods were employed for folksong-based appraisal of sorghum bioecocultural heritage.

### Focus group interviews

First, community based Participatory Rural Appraisals (PRAs) were made in 12 Farmers Associations (FAs) of highland, intermediate and lowland areas. Subsequently, participants were seconded by the community based on wealth and gender, to know how they ethnobotanically describe sorghum using folksongs. A total of more than 360 farmers in groups were interviewed. The selected *weredas *(*Wereda *is an administrative structure within the regional state comprising farmers' association, which is demarcated based upon agro-ecological and socio-cultural criteria) for this study were from the highlands: Girawa and Hirna; Intermediate: Alemaya and Hirna and from Lowlands: Babile and Dire Dawa. Either gender was considered when forming groups, but only one member from each household participated (Figure 1a and 1b). All the folksongs in the interviews were sung, recorded and discussed.

### Direct on-farm participatory monitoring and observation

One hundred twenty farmers were directly monitored on-farms, over the selected weredas in the three crop ecologies in order to assess the farmers' folksongs knowledge on sorghum. Songs were sung, recorded, and discussed in the course of on-farm monitoring.

### Key informant interviews

In order to assess the general folksong description of sorghum: key informants (Figure 1d), up to five per FA, MoA (Ministry of Agriculture) crop production experts, non-governmental organizations in each site were interviewed and songs were taped.

### Secondary data collection

The Harare Museum was visited for secondary data and inter-personal discussion with sociologists, museum curators and Harare historians in line with bioecocultural heritages of sorghum.

### Data analysis

Descriptive statistics, content analysis as outlined in [6] and interpretation were made.

## Results and discussion

### The people and sorghum

Historically, the study area which encompassed the Hararghe political administration, was influenced by various Arab Emirates (to 1875), Egyptians (1875–1885), and Italians (1936–1941). Owing to this fact, the dominant religion in the study area is Islam.

The major ethnic group in the study area is *Oromo *(95%). The others are *Somali *and *Amhara*. The eastern Ethiopia *Oromos *prefer to call themselves *Kottu. Kottu *means farmer in its broader context; Kottu are Islamised *Oromos *of eastern Ethiopia who make their living by working in agriculture. The *Oromos *have a complex social organization that operates based on *gossa*. Each *gossa *(intertribal grouping) lives in a village settlement called *ganda*, which is a conglomeration of 10 to 20 huts and a few *tikuls *comprising 60–100 persons. The *gandas *(Figure 1e) are located a short distance from each other. They are separated by a few trees mainly of *Euphorbia *which delineate these settlements from the farm fields. Seven major *gossas *were in the eastern region: *Ittu, Anmya, Alla, Babile *(Figure 1f), *Oborra*, *Jarso*, and *Nole *[7]. Except *Annya *and *Ittu*, the *Afran Kello *(the four sons of *Kello*) group make up the largest part of Harerghe *Oromo*. Among them, the *Kello *group, the *Alla *and *Nolle *are the largest [8].

Only a century and half ago, nearly 50% of the *Oromo *population of Hararghe were pastoralists. By the 1840s, only the Babile and the mixed *Oromo-Somali *groups to the east of Harar had adopted the agricultural practice of the *Harari *(commonly called *Adere*). The majority of the *Oromo *took up agriculture as the main occupation because of their participation in exchange relationships with the *Harari *who took up trade after the *Oromos *expelled them from their farmland. The *Harari*, who are believed to come from Hamasien (part of Eritrea), settled in Hararghe around the 13^th ^century [8]. Since their settlement, they have been producing sorghum as a staple crop and other various types of fruits (Pers. comm.). The *Harari *are the Semitic language-speaking inhabitants of the walled city of Harer. The Walled city of Harer which was built by Ali Nur, the cousin of Mohammed Gragň in order to protect the *Harari *from the surrounding *Oromos*, was recognized as a World Heritage site by UNESCO in 2006. The Harari people have been growing and eating sorghum as a dominant crop until very recently. The change in the means of living from agriculture to trading has also changed the use of food crops they eat from sorghum into wheat and *tef*. However, there are still some traditional dishes being eaten, which are prepared from sorghum only.

The context of agriculture in the region varies with ecology. The type of agriculture prevalent in the area includes crop farming, livestock farming, and mixed agriculture. The dominant food crops in the area are sorghum (*Sorghum bicolor *(L) Moench), maize (*Zea mays *L.), wheat (*Triticum aestivum *L and *Triticum durum *L.), barley (*Hordeum vulgare *L.), faba bean (*Vicia faba *L.), fieldpea (*Pisum sativum *L.), and haricot bean (*Phaseolus vulgaris *L.). Coffee (*Coffea arabica *L) and *Khat *(*Catha edulis *Forskk) are the major cash crops grown alone or in association with other food or cash crops.

Sorghum is the dominant food crop in the region. Farmers have been growing sorghum as long as they can remember; they estimated the time of cultivation to be at least or greater than 500 years (twenty generations), each generation being about 25 years. The farmers' history of sorghum farming is closer to the actual period of *Oromo *immigration to the region, which dates back to the 16^th ^century [8]. Moreover, in his visit to Ethiopia, Alvarez-the Portuguese, who travelled from 1520 until 1526, saw sorghum being cultivated in Ethiopia. As early as the 14^th ^century, the Egyptian, Ibn Fadl Allah Al-Omari referred to the cultivation in Ethiopia of wheat, barley, sorghum and *tef *and remarked that two harvests a year could be obtained [9].

Sorghum in eastern Ethiopia is grown from the hot and dry lowlands of 800 m asl (Figure 1g) to the cool highlands close to 3000 m asl (Figure 1h). It is thought that agriculture and thereby sorghum production started in the intermediate altitudes and then moved into both the highlands and lowlands. The reasons being that at times, the highland used to be very cold and covered with forests and the lowland had high temperature and infested with malaria transmitting mosquitoes; hence, unsuitable for human living and crop production. In view of the high population pressure in the intermediate altitude, the scenario has changed and farmers are moving up to the highlands clearing forests (Figure 1i) and to the lowlands for more cultivable land.

### Sorghum's strategic importance for farmers' livelihood

**Figure 2:**
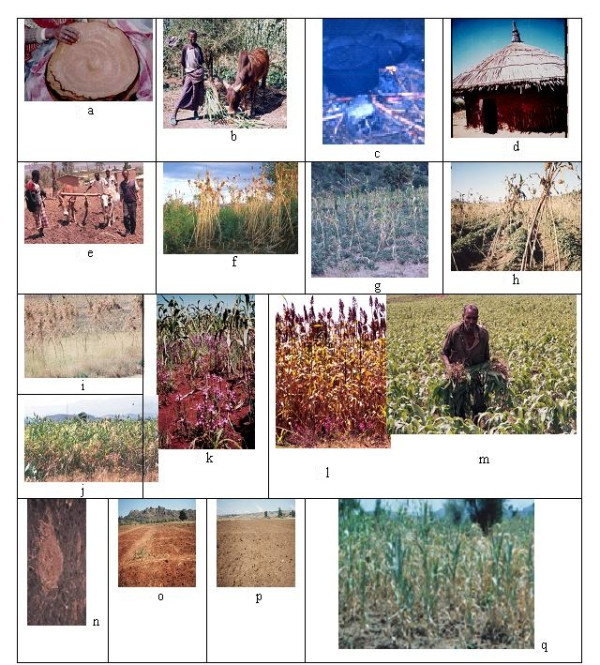
**2a. Sorghum *injera *(Ethiopian pancake) used for human food**. **2b**. A young boy feeding an ox with sorghum leaves and thin stalks **2c**. Sorghum for fuelwood **2d**. Sorghum for thatching of huts **2e**. Land preparation using *Guzza *oxen and labour sharing scheme **2f**. Sorghum-Khat alley cropping **2g**. Sorghum-groundnut mixed cropping **2h**. Sorghum-sweet potato intercropping **2i**. Sorghum with undersown wheat **2j**. Sorghum-maize mixed cropping **2k**. *Striga hermonthica *in Babile. Note the purplish flower colour. **2l**. *Striga asiatica *in Fedis. Note the reddish flower colour **2m**. Farmer thinning sorghum field, *Meta wereda*, Ethiopia **2n**. Application of organic manure on **Vertisols ***Alemaya Wereda*, Fendisha farmers association **2o**. **Regosol: **the dominant soils in the intermediate and lowlands **2p**. **Fluvisol **deposited soil, commonly found in the valley bottoms **2q**. Moisture stress. No seed setting due to terminal moisture stress at Miesso, 800 m asl.

Having co-evolved with the farmers, sorghum has been selected as a multipurpose cereal crop used for food, feed, fuel wood and construction. Sorghum is intimately associated with the daily lives of the farmers. Owing to these facts, farmers indicated that 'sorghum is everything for us', 'it is our food' (Figure 2a), 'it is our feed for animals' (Figure 2b), 'it is our fuel wood' (Figure 2c), and 'it is our construction material' (Figure 2d). It is the most important crop for the farmers' livelihoods and security [10]. A synthesis of the cropping calendar for various activities in sorghum is outlined (see Additional file 1).

Actually, the multifaceted benefits of sorghum for a present-day farmer are indicated in the following folk song:

*Dherate waaqa bahe alaati marfachisse*

*Kan du'ee Kute hinbanu bishingan*

*Gagaba bayfachissee*

'Sorghum is everything for every one of us;

Except for the dead ones'

As aforementioned, sorghum is a food, feed and tree crop in the region. As a food crop, various types of dishes are prepared out of it. The leaves, stalks, and chaff of sorghum are widely used as a feed crop for ruminants as grazing land is scanty and less productive. It is used as a tree crop by providing fuel wood and used as a construction material to build farmers' houses.

Sorghum's contribution to the household food security is described as follows:

*Himilali jartidha jarsa jollee dhiftee*

*Lagati qaceelte bola duwwa garanii buna*

*Dhalu dhageenyani*

*Manatti nadebissa jetee mangudo kadhate*

*Bishinga ashitu dhageenayni*

'In times of food scarcity in the highlands;

A lazy wife when the pit is empty of sorghum;

Though coffee is ready for harvesting;

She runs from her home down to the lowland;

However, when sorghum reaches for harvesting;

She will send an arbitrator to return home'

Sorghum is a strategically important crop for farmers' survival, livelihood and food security [11,12]. Sorghum needs to be present in farmers' pits throughout the year. In the absence of sorghum, conflict arises between a husband and wife, even if other crops are available to harvest (e.g. coffee). This is normally followed by a wife running away to her parents, in particular, in the hunger period that lasts from from June to September. As the time for sorghum harvesting approaches, an arbiter is sent by the wife to return home.

Farmers' determination for growing sorghum is indicated in the following verse:

'When a person gets sick due to God;

It is abrupt and the cause is unknown;

When he dies of God that is forever;

As far as we live, we will not stop sowing sorghum'

Sorghum is the most important food crop in the region. In Ethiopia, there are over 3,674,865 millions of farmer households dependent on sorghum production. In eastern Ethiopia alone, 635,342 farmer households are dependent on sorghum. Closer to 90% of the farmers are using the varieties developed by the community [11]. These varieties have been grown on-farm for hundreds of years. These sorghum-growing traditions still continue in the current generation.

The comparative and higher values of sorghum *vis-à-vis *other crops for farmers are very much praised. A man who has sorghum in times of trouble is always praised as follows

*Oboraan makka taatee itun madina taatee*

*Madinan afranqallo gurba waaqabu ta'tee*

'Abera whether he is in *Meka *or *Madina *or in *Itun*;

The four children of *Kello *in Hararghe;

Those with sorghum are always better off'

The majority of the farmers are Muslims. It is common to go to ***Meka ***or ***Medina ***(holy muslim sites in Saudia Arabia) for praying, blessing and also working. As Saudia Arabia is an oil-rich country, it is expected that people who went there will be rich. The farmers in Hararghe still growing sorghum are expected to be far richer than those in Saudia Arabia. This is to show the crucial importance of sorghum for farmers' livelihood and survival.

When the farmers praise sorghum they use the following expression

*Amma jetuus dalanee manggistwen mana dhabee*

*Gebryiee jechu male maqa kee nin walale*

'I do not dare to call sorghum with the name *Bishinga*;

I just call it ***Gebryiee ***(*adoring name of praise for sorghum*) for all its importance.

Sorghum is locally named *Bishinga *or *Mishinga *[5]; due to its multipurpose values, farmers prefer to give it an adoring name such as *Gebriyee*. They believed that calling it by its common name might de-value its importance.

### The bio-socio-ecology of sorghum production, management and utilization

Based on thermal and moisture regimes and length of growing period, Ethiopia has 18 major agro-ecological zones [13]. Most of these agro-ecological zones are also found in the study area. These range from the hot to warm arid plains of the *Ogaden *lowlands to the cool humid highlands of the Chercher Mountains. The rainfall ranges from 400 mm to 1800 mm per annum with monomodal and bimodal rainfall pattern. The temperature ranges from 10°C to 35°C. LGP (Length of Growing Period) ranges from 90 days to 240 days.

Hence, sorghum is grown in the region with various thermal and moisture regimes which constitute different LGPs. The tremendous agro-ecological variations in the region have resulted in high on-farm genetic diversity for sorghum. Long, intermediate and short cycle sorghum is grown in the highlands, intermediate and lowlands, respectively. The longer cycle sorghum is normally planted in the time of the *Belg *(short) season (Feb-March) and continues to grow to the end, Nov-Dec, of the *Meher *(long) season. Hence, crop failure in the *Belg *season affects the long cycle sorghum. This is common in the highland and intermediate areas, which normally results in drought, but it does not happen in the lowlands where short maturing sorghums are commonly planted around the beginning of the *Meher *(May-June) (see Additional file 1).

#### Land preparation

The topography of the study area includes the undulating hills and bottom valleys (Figure 2e). Farmers used locally made and available farm tools for cultivating their sorghum fields. This is predominantly done by men. To prepare land, wealthy farmers use tractors while poorer farmers employ oxen using *Maresha *(a traditional metal tipped wooden plow), manual digging is done using *Dongora *(a pointed-tipped stone-capped wooden digger followed by land levelling) and water harvesting structures are worked using *Akaffa *(hoe/shovel). The most widely used method is manual land preparation. The well-developed labor and oxen-sharing scheme *Guzza *is used in the course of land preparation (Figure 1e) encompassing inter-cultivation, harvesting and threshing.

When '*Guzza*' was performed with a farmer whom they are not happy with, the following verses reveal their sentiments:

Hama oolu nifunee ulule lafa fune

Waldhabnee fafacaanee hala sayo badiidha

'Talking, the farmers who came for '*Guzza*';

While we went to the farm, we were fluting;

Suddenly we were reminded of the bad character of the farmer;

Then we went back and dispersed'

The participants do not perform *Guzza *for any farmer, as the social profile of the farmers is considered for this resource-sharing scheme. When a farmer who is not accepted by the community asks for *Guzza*, the farmers will not be willing to participate and *vice versa*. After *Guzza*, if farmers who participated were not satisfied with the host farmer because they were not thanked adequately they expresse their dissatisfaction as follows:

*Alkadiun hindubane olin dubin nungenyee*

*Danbooba gona male sa'abo faarun hingenye*

'Amidst the farmers Kedir did not utter a word;

Or no message from the hosting farmer;

Though done very well;

He is not thanking us from his heart'

It is very common for the host farmer to thank collaborating farmers for sharing labour and other inputs. The form of thanks is also expressed by hosting a feast after the work is finished. The lack of a gratifying feast after *Guzza *displeases the participating farmers.

*Guzza *is the major labor association. In this arrangement, 10 to 20 men are involved in helping the host farmer in weeding, harvesting, and threshing and sometimes in house building. The host normally prepares food for the participating farmers. Similarly, the wife of the host farmer is helped by the wives of participating farmers for the preparation of meals such as *Honqua *(roasted grain), *Hoja *(a drink made from coffee leaves and mixed and boiled with milk served after meal) and *Khat *(a mild drug crop chewed for its stimulant leaf) for lunch; *Injera *(pancake) and *Genfo *(porridge) for dinner. *Guzza *will rotate to other farmer, which is called as *Fereka*. Other resource sharing associations called *Merro *and *Afosha *involve lesser number of farmers, where exchange of labour and animal power is involved.

#### Seed selection and preparation

Men, women and children are involved in seed selection. Both intra-varietal and inter-varietal seed selection are made by the farmer in order to meet their biophysical and socio-economic needs. In the course of seed selection, they use various criteria for genetic enhancement. The criteria used for seed selection includes morphological traits (leaf number, basal tiller, stay green, plant height, threshability), biotic stress related traits (reaction to weevils, stalk borer resistance), abiotic stress related traits (drought, frost, low soil fertility) and use related traits (for preparation of various dishes, total biomass production, feed values, stalk marketability). The rich selection experience on the indigenous crops such as sorghum is also applied to other crops; namely, maize, bean, potato (*Solanum tuberosum *L.), which were recently introduced to the area [14,15].

#### Sowing and cropping system

Sorghum is commonly sown by hand either in broad casting or in row planting. It can be one variety or a mixture of varieties sown. Depending on the ecology of sorghum production, the planting time varied. The range of planting time spans from mid March to mid June (see Additional file 1). It is alley-cropped with coffee and *Khat *(Figure 2f), intercropped with bean, groundnut (Figure 2g) potato and sweet potato (Figure 2h) and mixed cropped at various stages with cereals such as wheat (Figure 2i), maize (Figure 2j), barley, and *tef*.

Farmers indicated the critical importance of time of sowing that a farmer has to operate appropriately within the period, if missed, the season is lost which signified that sorghum farming has been predominantly rain-fed.

*Addun dhiitee galgala loon gale mitijaba*

*Addun dhiite hindebitu yabo xiqoon harka saama*

'The time is now approaching for sunset;

The livestock are going back home;

The sun set will not rise today;

And hence let us work hard timely'

The rainfed agriculture warrants the need for implementing the cropping calendar (see Additional file 1) appropriately. The absence of relentless effort affects the whole farming operation and puts the household food security at risk.

Of course, sorghum is also used in their daily expressions that people have to work to live. In the following verse, the crow is used in place of a lazy farmer.

*Bishingan bishinguma malifi midhaan*

*Nama nyata Ya' Qura*

*Hojodhu buliimale molifi harka nama*

*Laalta nadura*

'Oh you crow, you crow;

Sorghum is sorghum;

Do not see the hand of others;

You have to work to make your living'

It is traditional in Ethiopia to help each other. This is particularly common when the farmer needs assistance. However, a farmer who is capable and has enough resources such as land and labor, is not supposed to be helped.

#### Weeding

Weeding is one of the labor-demanding tasks in the area. Manual weeding is the common one. Only very few farmers use herbicides. Weeding is done two to three times per season (see Additional file 1). Intercultivation (additional file 1), sometimes called *shilshalo*, is used for weed control and for preparing the land for fertilization and undersowing of legumes such as beans, too. The most important weeds are *Striga *(Figure 2k and 2l) and *Parthenium hysterophorus*. *Digitaria scalarum *is also an important weed. However, most of the non-noxious weeds are used as animal feed.

#### Thinning

It is one of the most important farmers' field and varietal management measures practised (Figure 2m) at various crop growth and developmental stages (see Additional file 1). It is done for three reasons: Firstly, to optimize plant stand by thinning out from over populated spots and transplant to part of the land with less population. Secondly, to remove diseased, insect-affected and poor looking ones. It is negative selection for poorly performing plants. Thirdly, to remove out plants that are not flowering or seed setting. Hence, resource competition is minimized. In general, all the thinned out plants at the right stage will be transplanted; if not, they will be used for animal feed.

#### Fertilization and inter-cultivation

Farmers normally use organic fertilizers (Figure 2n). The use of inorganic fertilizers, namely UREA and DAP, has increased very recently due to the agricultural extension-promoted package program. Usually 50–100 kg/ha of UREA and DAP is used by the farmers. However, farmers complain that their land is already 'badly programmed for fertilizer' and there is a decline in the land productivity since the start of chemical fertilization. In addition to this, the low purchasing capacity of farmers and increasing cost of fertilizer has resulted in decreasing its use on sorghum. Besides, farmers normally prefer to apply fertilizer on commercially important crops like *Khat *and coffee than on sorghum (see Additional file 1). This is because of the higher market value of *Khat *than sorghum [12]. However, as most of the farmers' varieties are selected and grown under low input conditions, they are expected to be low soil fertility tolerant. Actually, farmers have noted the differential soil fertility demand of some varieties as compared to the others.

Farmers know very well what care has to be given for sorghum following each stage of development

*Gabrriyeen bala busse biyeedhan iti bussa*

*Fayya dhaii madhabda ajajaan itti bussa*

'Sorghum (*Gabrriyeen*) has grown up;

It has stretched its leaves;

It needs soil heaping;

Encourage me to do it'

Sorghum being an established crop, farmers knows very well what care has to be given following each stage of development. After the unfolding of the first leaves, soil heaping is done for proper growth and development of the crop.

#### Water management

Sorghum is grown from the dry lowlands of Meiso, Babile, Dire Dawa, and Goloda to the high rainfall and cool highlands of Girawa and Hirna. The ecological ranges in which sorghum is grown has resulted in having tremendous variation in available moisture. The variation in rainfall over the study area ranges from 400 to 1800 mm rainfall. Sorghum grows in different soil types (Figure 2n, 2o, 2p) ranging from heavy soils in the highlands to the sandy loam soils in the dry lowlands. It is also adapted from high acidic soils in the highlands to the saline-sodic soils in the lowlands. In view of the range of sorghum growing ecology, it faces various stages of moisture stresses; namely, initial, intermittent and terminal stresses (Figure 2q). However, the known farmer indigenous technical knowledge on making soil-water bunds has helped in harvesting soil moisture for crop growth and development. Farmers give emphasis for both high yield and stability [16,17] because of the wide environmental variation in sorghum growing varieties. Indigenous soil and water harvesting practices associated with the drought tolerant nature of the crop, varietal mixture and appropriate planting date helps farmers to manage soil moisture efficiently for the crop. Farmers' appreciation of the drought tolerance of sorghum is indicated as follows:

*Dheeratee hawa dhaqee alaati marfachiissee*

*Gonbobee karaa dhaqee imaltu marfachiissee*

*Kan du'e dhabamee Bishingan abba issa gamachiissee*

'Due to drought the farmer lost hope;

Sorghum fallen on the field blocking the farm roads;

Was surrounded by crows;

It received little moisture;

It then resurrected and pleased the grower;

The farmer joyed and harvested the crop'

Sorghum is one of the leading drought-tolerant crops. It is considered as the 'camel crop of cereals' for its drought resistance and recovery behaviour (Figure 1h). When all other crops fail, sorghum persists to survive in times of moisture scarcity. In the smallholder farmer level, it is used as a risk aversion and strategic crop for meeting household food security. This is also indicated in the next song that a sorghum farmer who lost the crop is analogous to 'a man in a sunk ship'.

In the course of a complete crop failure, farmers expressed the graveness of the situation as follows:

*Ilaalkoo bakaa jarssa gangeen badeenii*

*Gurbaan dalagee dhabe nama doniin cabxeenii*

'A mule has got rid of an old man named ***Ilaalko***;

A farmer that completely lost sorghum harvest;

He is comparable to a man in a sunk ship'

#### Disease and insect control

**Figure 3:**
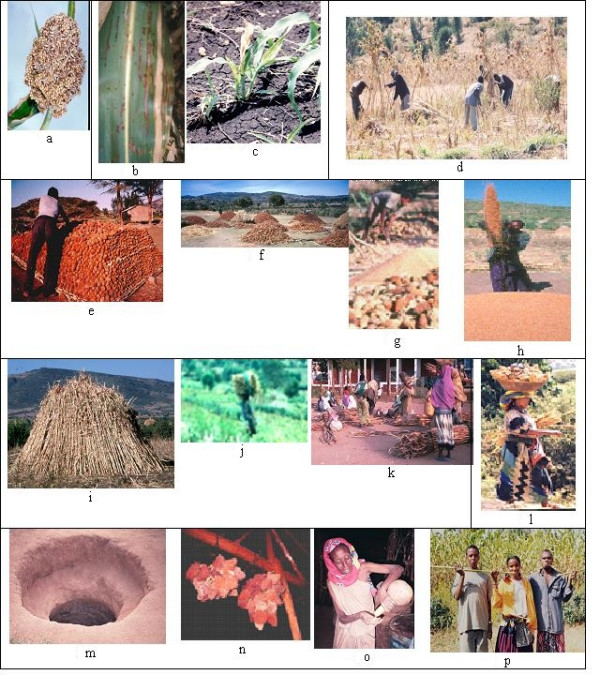
**3a. Kernel smut (*Sphacelotheca sorghi*)**. **3b**. Leaf blight and Bacterial stripe **3c**. Shootfly damaged plants. Note its effect on the drying up of the central leaf **3d**. Harvesting with *Guzza *labor sharing scheme. Before harvesting is done, the stalk are bundled to reduce lodging and for ease of harvesting **3e**. Heaping sorghum on the threshing ground **3f**. Heaps of sorghum of the village in common threshing ground **3g**. Manual threshing **3h**. Winnowing **3i**. Piles of sorghum stalks **3j**. Stumps removed and transported by woman for fuel use **3k**. Sorghum stalk market at *Alemaya *commonly marketed by women **3l**. A woman farmer carrying sorghum panicles for seeds to the house **3m**. Pit for storage inside the house 3n. Seed panicles hanged on the roof over the kitchen 3o. A woman farmer showing germplasm storage for long period in a bottle guard. 3p. School boys and girl chewing sweet sorghum stalk

Sorghum in the region is affected by various types of insects and disease. Major diseases are smut (Figure 3a), mold, ergot, downy mildew and other bacterial diseases (Figure 3b). Major insect pest are stem borers and shoot fly (Figure 3c). Farmers use cultural, botanical and chemical methods for crop protection.

#### Harvesting and threshing

Sorghum is harvested when it reaches physiological maturity, the stalks are bound, leaves are defoliated and belled. Normally harvesting is started after drying the stalks for a while (Figure 3d). Then farmers start cutting sorghum with a *Mencha *(a long-handled locally made tool for various agricultural activities) or sickle, for cutting the stem at the base of the plant. Then the panicle will be cut immediately with the same tools after harvesting or cut after letting stalks dry for some days. Then the cut panicles will be transported and heaped on the threshing ground (Figure 3e and 3f). This is followed by trampling of sorghum heads using heavy and thick sticks (Figure 3g) or livestock (oxen, donkey, horses). Finally, it is winnowed (Figure 3h) against the wind, put in a sack and transported to the house.

Of course farmers appreciate the importance of sorghum for their livelihoods and give due attention for managing and harvesting sorghum carefully.

*Muraa je'ee maraa irkisa je'ee*

*kan jiraa marse nagsse digisaa jedhe*

'Oh collect sorghum carefully;

Pile up the stalk vertically;

Heap up the panicle;

Acclaim sorghum the same way you do for the heroic'

*Garyeen gabra bitee enyutu syati nugessa*

*Udatu ta'eti bishingaan qalbi nujabessa*

'Sorghum always encourages us;

While it is heaped on the threshing ground;

It pleases our heart;

And hence no need for livestock farming (or no need to take care of livestock)'

Farmers become happy when they start harvesting. Actually, there is nothing left behind after harvest; every part of sorghum is used for various purposes. In view of this, due attention is given to harvesting every plant part.

#### Residue management

As aforementioned, sorghum is a multiple value crop. The different parts of the plant are used for many purposes. There is nothing plowed back to the soil. The leaves are for animal feed, the stem is for fuel and construction and stumps (Figure 3i) are removed and used for fuel. The stem is piled and stalked for a few seasons to be used as fuel (Figure 3j). Sometimes farmers get better prices in winter by selling stalks for fuel (Figure 3k) than selling sorghum grain in the market. The way the residue is collected and managed indicates how the livelihood of farmers is attached with sorghum.

#### Grain and seed storage

Panicles selected for seed (Figure 3l) and threshed grains are taken to the house. Grain and seed are stored in various ways. They are commonly stored for one season. Pit (Figure 3m) and hanging panicles (Figure 3n) over the roof are dominant storage methods for grain and seed respectively. Some farmers store seeds for long-term storage in bottleguards (Figure 3o).

#### Consumption

Most farmers grow sorghum in the region for the household consumption. Only some of the farmers are growing sorghum for both sale and household consumption; 90% of the farmers produce sorghum for household consumption. As farmers indicated, it used to be the only cereal crop in eastern Ethiopia, except when other cereal crops such as maize, barley and wheat were introduced to the region in the last 100 years. Sorghum is consumed widely as '***injera***' (traditional fermented pancake); bread, '***Shummo***' (boiled grain), '***lafis***o' (sorghum powder mixed with fenugreek and milk), and '***Honqua' ***(roasted grain). The sweet sorghum types are also eaten green as '***Eshet' ***(row grain) or roasted as '***Enkuto' ***(roasted panicle). Traditional alcohol drinks such as '*Tella*' or '*Areke*' are also made from sorghum by other region farmers. There are specific varieties identified for various types of consumption [5]. Canes of sweet types of sorghum are used as a substitute for sugar cane (Figure 3p).

The satisfaction in the frequent consumption of sorghum in the household is appreciated as follows:

'A wife and husband quarrelled;

Because they do not have sorghum;

However, they slaughtered beef;

Not possible to eat meat continuously;

But possible for sorghum'

Sorghum is the staple crop in the region. It has been used as food crop since domestication. Various types of dishes are prepared from it. The previous song indicates the role sorghum plays in the household food security. Even if meat is in the house, it cannot be consumed year in and out like sorghum. However, farmers are pleased with eating sorghum continuously.

Sorghum is also associated with the daily life of the young people

*Addun dhiitee galgala loon gale mitijaba*

*Addun dhiite hindebitu yabo xiqoon harka saama*

'A young man that eats green sorghum;

Is always appreciative of the beauty of girls;

And said "the foot takes me where the heart is"

As an intimate crop with the life of the farmer at all ages, when the crop reaches the early dough stage, it is the time for the youth to search for their mates. Consumption of sweet green sorghum is commonly associated with sexual excitement. At early stages of sorghum physiological maturity, most of the work is over except harvesting and threshing, and hence plenty of time is available for amusement.

Farmers also appreciate the blessing from God;

*Walisaan imaltawee g'adhe waraani senee*

*Aka wadaaja bulee gabro wa'nurii seene*

'The singer stood up for singing;

Then youth felt heroic;

When I implore God;

He gave all his blessing on sorghum and it become abundant'

Comparing sorghum with cows milk;

*Gororan hidaa issatibaali libasha issati*

*Anani saya fakaata bishaan fedaga issati*

'Sorghum stands by its foot;

Wears its leaves;

After dehulling in a pestle;

What comes out is like the cows milk'

This indicates one of the traditional aspects of processing of sorghum. Dehulling, though not used on a large scale, is partly done for the preparation of some local dishes. After dehulling and pounding the white endosperm type of sorghum variety, it is closer to milk in both colour and nutritive values.

In times of food deficit, the importance of sorghum is indicated as follows.

'The past hunger period has affected me;

The neighbouring farmer stood beside me offering no help;

But if sorghum was around I would have been saved'

This happens commonly when there is a late start of rain in ***Belg ***(short rainy) season or early cessation of the ***Meher ***(long rainy) season. When farmers do not have any reserve grain, then the effect will be prominent.

### Folksong-based characterisation of folk genera, species and varieties of sorghum using folksongs

At the level of folk genera level, farmers describe sorghum as follows:

'Leaf number is less than twenty;

Panicle holds thousand seeds;

A clever farmer takes hold of it'

Sorghum in formal taxonomy is described as a cereal plant with a range of leaves numbering 14 to 17 and having a panicle length (4–25 cm) and width (2–20 cm) and seeds per panicle numbering from 500 to 3000 [4]. This clearly showed that the folksong-based characterisation is comparable with the scientific ones.

### Comparison of sorghum with other cereal crops

1. Comparing the nutritional values of maize and sorghum. The higher nutritional value of sorghum as compared to maize is well explained by the farmer as follows:

The one who eats maize is '***Kera***'

But that who eats sorghum is '***Ala***'

'*Kera*' refers to insipid sorghum types where the stem is easily broken and it is common type of varieties that are susceptible for lodging and had less multipurpose values. For example, *Wegere *folk species. However, '*Ala*' refers to sweet stalk types, lodging resistant, very strong and higher multipurpose types. For example, *Fendisha *folk species.

When comparing sorghum and maize nutritive value farmers sing:

'Sow sorghum and maize together;

Maize is for selling and sorghum is for eating'

2. Comparing sorghum and wheat:

'Seed of sorghum looks like wheat;

And those that do not have sorghum are unstill'

*Qamadiin sinde ta'tee sinden malaqa'tee*

*Gurba qamadii qote ki'yoon malaqa ta'tee*

'The one who has sown wheat, he has money;

But he must stop thinking about ***injera***'

This indicates that sorghum is used more for household consumption than as a cash crop while wheat is used as a cash crop.

3. Comparing sorghum and barley:

*Bishingan dada jiraa garbun imana saarii*

*Issatun sirra dhaama sasaba marimaanii*

'Oh sorghum be adorned

Barley is for cash

But sorghum gives all the strength and satisfaction'

The comparison of sorghum with maize, wheat and barley showed that sorghum is predominantly grown for household consumption and the other crops for sale. The major cash crops in the region are *Khat *and Coffee.

### Associated folksongs for the folk species and varieties

#### Muyra

*Muyra *is a variety that has been grown by farmers for many decades. Farmers have been crediting *Muyra *as follows:

'From your children, trust your daughter;

And from sorghum varieties, trust ***Muyra***;

Take care of your goat, ***Muyra***, camel and your daughter;

As sons and other livestock do betray you'

As per the farmers' description, *Muyra *is one of the most stable varieties. Hence, when other varieties fail to grow, *Muyra *will grow to yield and please the farmer. This is analogically compared with camel from the livestock and daughters from children who are loyal in testing conditions.

For White *Muyra*:

'The stalk is sweet;

When threshed it is free of glumes;

When dehulled it looks like milk'

One of the most important panicle traits used in folk classification of sorghum varieties is their threshability. There are three classes of threshability; namely, freely threshable (0–10% unthreshed kernel), semithreshable (10–15% unthreshed kernel) and difficult to thresh (>50% unthreshable). ***Muyra ***is classified in the freely threshable group.

Describing sorghum (e.g. ***Muyra***) throughout the growth and developmental stages:

'When it starts growing, it looks like ear of camel;

When the hail comes, it looks like a person with torn clothes;

When it gets little rain after drought, it recovers quick and resembles a satisfied rich man;

In times of booting, it looks like a pregnant woman;

After booting it looks like face of a slave;

After seed setting it looks like the face of a girl'

There are three main phases of sorghum growth and developmental stages. These are vegetative phase (germination and seedling development), reproductive phase (infloresence development and fertilization), and maturation phase (seed development). '..it looks ear of camel' and 'the pregenant women' describes the vegetative phase; 'face of a slave' describes the flowering stage; 'the face of a girl' describes the seed maturation phase.

Growing sorghum in tropical ecology faces torrential rain, sometimes hail and complete cessation of rainfall. Farmers described the response of sorghum for both hail and drought. In times of hail, the leaf area is decreased due to damage and in case of drought, sorghum responds by folding in leaves and reducing growth rates. Immediately, when it receives rainfall/soil moisture, it unfolds its leaves and grows well.

Women recite songs while transporting panicles from the field to the threshing ground. To show the panicle size of the varieties

**'*Muyra ***is angry;

***Fendisha ***is angry;

The container is incapable to carry'

One of the roles of women in sorghum production is to transport panicles from the field to the threshing ground (for grain types) and to the house (for seed types). Normally transportation is made by donkeys or humans. When women transport, they use an open container made of grass and put it on the head. *Fendisha *and *Muyra *varieties have large panicle sizes. In order to describe the large panicle size only few panicles (upto 15) of these varieties can be carried compared with other varieties.

**'**Ah ***Muyra ***Ah ***Muyra***;

With out you I am helpless;

When I have you, my soul gets pleased'

#### Qille

Describing its panicle morphology:

*Bishingan bishinguma hunda Quleetu Caalee*

*Naanno dubra fakkate itiin Sirbuukajeele*

'Ah sorghum named as *Qille*;

Your head looks like shurube (tightly plaited hair) of a girl'

Describing its full-season maturity

*Qi'lee facaafatu Yakatiti qophawii*

*Shanyii alati bassii furmata kee*

*Kadhahu Rabbii itti Harobufii*

'If you need to get harvest from *Qi'lee *variety;

Be ready to plant in February;

Then beg for God to have rain'

Based on maturity classes, sorghum is classified into early, intermediate and full season types. In the case of full season maturity types, such as *Qille*, planting has to be made in February (beginning of short rainy season) and it stays until October. This showed that rainfall is needed throughout the growing season; when it does not rain sufficiently farmers indicate the need to implore God for rain.

#### Fendisha

Appreciating the excellent quality of *Fendisha*

*Ashitan Fendisha ni midhaga*

*Nawadi nashukunihin jedhu*

*Ilkeen irra dugugata*

*Qamatu sigabbata*

*Fendisha *how wonderful is seed

Because it does not demand roasting

Because it threshes easily

Because it fattens the body well

*Fendisha *is one of the sorghum varieties developed by the farmers and is highly demanded for its threshability, good quality seed and better nutritional composition.

#### Bullo

**'**The one with ***Bullo ***is happy;

The one without it is vulnerable'

***Bullo ***is a variety grown in moisture limited (dry lowland) areas. Farmers in this ecology, who do not have ***Bullo***, will find it difficult to survive.

## Ethnomusicology analysis of folksongs

A description of folksongs associated with each sorghum farming activities have been given in the preceding pages. A general analytical view of the folksongs from the perspectives of ethnomusicology is provided in this section of the paper.

### Folk songs as a part of folk literature

The lack of comprehensive studies in folklore of Ethiopia has constrained this study. The collection and publications of oral literary material by Ethiopia begins with *Belaten Geta Hiruys's Masehafa Qene *(Book of *Kene*) a collection of *Geeze *poetry in 1918. The Ethiopian oral literary works begin to appear in European Journals, magazines and newspapers in the course of extensive studies by Europeans of African linguistics to aid colonization and evangelization [18]. Though Wolf Leslau mentions as earlier collections, *Yalakso Zema getem *(dirge poems) [19].

Those studies focused mostly of the northern Ethiopia where *Tigringa *and *Amharic *Semitic languages are dominant. This study, however, was conducted among the *Oromo *ethnic group in eastern Ethiopia. Oromo is a Cushitic language.

Folkart or folklore in general encompasses oral literature, material culture, and custom and festival. Oral literature is structured on a sense of community and holistic view of togetherness of man, nature and culture [20]. Oral literature in general includes folk narrative and folksongs [20,21].

Folksong as a part of folk art or folklore are examined for the first time for describing the bioecocultural heritage. Folksongs described from land preparation until consumption covers the humanistic, sociological and psychological perspectives. In the humanistic perspectives, it shows the creative role of the narrator or bard and the artistic role of the folk, the sociological perspectives emphasized cultural norms and values; and that of the psychological perspectives focus on the behavioural pattern in the folksong in describing bioecocultural heritage.

Important aspects of folksongs are the association of the text with tune, requiring the folklorist to trace the melodic as well as the textual history. A major division separates the ballad that embodies the narrative and the lyric that express the emotion [22]. Most types of folksongs described include ballad stories, for instance, those describing the varieties are in these groups.

### Origin of folksongs

The origin of the folksong is not clear, however. None of the folksongs recorded are associated with myths or rites; rather they are associated with the reality of sorghum farming. In many other folkarts, the mythical or ritual origins are not uncommon. One thing that is implicit during the study was folksongs' anonymity. None said that folksongs belong to individuals and are credited to someone.

There are folksongs recited in various social strata; namely, women, men, old and young people. Some of the folksongs are popular in all social classes. However, it was noted that in the different *weredas *surveyed there are few people who are well versed and popular with the folksongs in each village where the community has given the *de facto *recognitions. These people repeat and remember what they have heard and recite for the people, although some additions, deletions, innovations or modifications can be made. Despite the popularity of some people for the folksongs, anonymity of them is accepted regardless, though. This is in contrary to the contemporary trend in the process of individualisation where in case of folksongs they are public property.

### Folksongs: importance and function in bioecocultural heritage

The major purposes of the folksongs in describing the bioecocultral heritage can be summarised as follows:

1. Oral transfer of knowledge, practise and technology related with sorghum. Diffusion with integrity and consistency with its major tenets intact is expected; though, the need to contextualize with changing socioeconomic and bioecogeographic environments is not uncommon. Acculturation in folksongs though not predominant, the presence and coexistence of three major ethnic groups in the farming community namely, the *Oromos*, *Somalis *and *Amharas*, do not mutually exclude from happening. The diffusion and acculturation from one region to another is enhanced by traders, migration and movement of people and participation in the various social functions such as wedding and funeral ceremonies. The transfer of the folksong is purely dependent on the memory of the speaker and singer. In the transfer of folksong, narrations come from the old people to the children. The old people had heard them from ancestors though they sometimes modify, innovate and add. If it is acceptable to the listeners, the song will be repeated and transferred to generations. Hence, documentation of the folksongs in this study has been initiated, as written literature is better than the oral literature from heritage points of view. This is in agreement with the idea that folksong is transmitted orally by all or most members of a culture generation after generation; it represents an extremely high consensus about patterns of meaning and behaviour of cultural rather than individual significance [23].

2. Teaching the successive generations on the culture, management and use of sorghum.

3. Folksong covers diverse themes from seed preparation up to the utilization of sorghum.

4. Symbolistic description. The nomenclature, classification and criteria of folk taxonomy are described [5]. The folksongs have also partly described adaptation, maturity, characteristics and use of the varieties developed by the farmers. On folk genera level, for example, folksong that describes sorghum is '*Leaf number is less than twenty; Panicle holds thousand seeds; A clever farmer holds of it'*. This is in agreement with the assertion that song language is often more permissive that ordinary speech, music can answer questions which if asked directly would probably not be answered, or only with the greater reluctance [24].

5. Mobilization and organization of the farmers: the use of *Guzza*, a sharing of labour and other resources is indicated. The presence of folksongs preceding and accompanying *Guzza *helped to mobilize, organize and motivate the participating farmers.

6. Depiction of the social norms: The norm in which the society has to accept has partly been illustrated by folksongs. Folksongs are both as young and as old as man depicting the various norms over a period of time that is accepted across the communities.

7. Describing the farming system: three ways of interactions of farmer-sorghum-environments are also portrayed, that is., how the farmers manage, produce and utilize sorghum in specific environments.

8. The folksong serve as a process of socialization

9. They are used as a means of entertainment

### History of Ethiopian folk music

It is known that Ethiopian music was developed in the time of Saint Yared in the year 600. Saint Yared was the composer of Ethiopian Orthodox Tawahado church and prepared the signs that are used as musical notes. This is comparable to that of musical sign of Catholic Church song developed by Guirdo Da-Ertho, from 950–1050.

Ethiopia is at a crossroad of Africa, Middle East and Asia which resulted in the mix and share of cultures and norms. Ethiopian music is understood as the African "East Horn" which is more influenced by Islam [25]. Ethiopian folk music *color *is stressed more than either *progression or harmony *[26]. Anyone listening Ethiopian music should not expect to be rewarded with finding music of structural, harmonic or rhythmic subtlety; but there is much else of great values and interest to be revealed-infinite melodic variation, strongly emotive tunes and verses that spring directly from the volatile eager spirits of the people, and links the past that may well prove to throw vital illumination on the cultures of other Middle eastern peoples [3]. It is commented that Ethiopia is located so to speak at the very crossing point of the important musical influences: looking at the map position of Ethiopia versus Africa Middle east and Asia, it is speculated that in this area the North-African arabesque chromatic style had to meet the pentatonic, or even prepentatonic musical world of central Africa [27]. Indeed, the musical material reveals the fruitful juncture of the two different musical styles and even more than that the surprising Central and East African relations as well as the neighbourhood of Mediterranean Europe.

Despite the aforementioned speculations, the geographical position has not disallowed the country from having unique culture, music, religion and norms. Ethiopia has over 80 ethnic groups with their own languages, diversity, culture, religion and norms. Hence, it is expected that the musical styles will be different. The diversity of peoples found in Ethiopia only adds to the confusion surrounding certain aspects of the music. Of the 80 ethnic groups, only musical setups of three to four are partly studied. Even for the one studied, the available information is not sufficient. Hence, there is no one kind of music that could truly represent the entire country, as the country is endowed with so many ethnic groups and languages, besides its geographical, political, and cultural isolation.

### Ethiopian musical instruments, modes and analysis of collected folksongs

The dominant musical instruments in Ethiopia are *şä*na*ş*el (sistrum); M*ä*qwamy*ä *(a T-shaped metal head with wooden or metal stand); *Kabəl *(a pair of rectangular woodblocks); *Dube *(a single-head bowl shaped drum); *Kabaro *(the conical drum); *Kirar *(a five- or six-stringed plucked lyre); *Masinqo *(a single-stringed bowed spike fiddle with a diamond-shaped sound box); *Bägänna (*a ten stringed plucked lyre); *Wašänt *(a hollow bamboo end-blown flute with four finger holes vary from 30 to 75 cm in length); *əmbilita *(is a set of three end-blown flutes). Further details about the Ethiopian musical instruments can be found in [20,21].

*Qəñ ət* is comparable to the English musical term *mode *[21]. Many *Qəñ ət* exists but no study has been devoted to those of different ethnic groups. The *Amhara Qəñ ət* has at least four: Ančihoye and *Təzəta* are the two oldest modes mean *you are my love *and *my dearest one respectively*. *Ambasəl* is derived from *Ančihoye *and *Bati *from *Təzəta*. Ethiopian music has similarities with that of other cultures. The *Ambasəl *mode seems to be found in Japan [21]. The *Təzəta* mode is found in China [25], the *şänaşel* or sistrum was found in Egypt [27]; lyres resembling the *Kirar *are found in northeastern Africa [28]. When notated on a paper Ethiopian music appears frozen and featureless, but what we actually here is an unending variety of changes and fluctuations, and fine distinctions difficult to capture on a paper [20,21].

With all the limitations discussed above, a comparative folksong analysis has been made for the recorded ones with standard parameters of music (Table 1). However, this is not exhaustive by itself but should be used as a stepping-stone for full-scale study of the ethnomusicology of *Oromo *folksongs.

**Table 1:** Analysing Oromo folksongs* describing the bioecocultural heritage by music parameters

*Parameters*	*Oromo folksongs*	*Remark*
Lyrics	More than one issue is addressed	With the exception to some of the folksongs, most of them describe various and more than one issues

Vocal style	Less ornamental to ornamental	Sometimes melismatic or strophic forms exist

Timber	Soft to less harsh	Occasionally strident and forced singing

Performer arrangement	Responsorial-leader chorus alternation is the dominant one	At times chorus-chorus alternation style. Responsorial-leader chorus alternation of male songs is less often followed by acclamation of women group. Few Solo songs exist.

Melody	Have a narrow range up to a fifth interval; melodies are patterned in descending and ascending order; 2 or three melodies are used	The melodic form of the folksong is strophic consisting of two to eight lines. The relationships among the lines do vary or are similar. Some of the folksongs have similar or different content. Most of the folk songs are monophonic and some are polyphonic (more than one melodic line). Most of the songs listed here for describing the bioecocultural heritages are sung without accompaniment of instruments and hence are predominantly vocal folksongs.

Scale	Major; natural minor; pentatonic major	Most of the folksongs collected here are diatonic or pentatonic or prepentatonic

Rhythm	Hard to identify; not a major element; 3/4 or 6/8	The rhythm and metre are not similar throughout the folksongs described. Mostly they are non-metric as they are mostly vocal. The music has been described as primarily melodic with simple rhythmic accompaniments that can be similar or different.

Song type	Both secular and sacred	Folksongs are impulsive creations which are not developed by artists in organized ways hence can have various foci.

QƏñƏt*	T*ƏzƏta *and *Bati*	Most of the folksongs are composed without notations. It might be the same composer or different who created both the words and the music. One of the major classes of the folksongs, the ballads are narrative songs for describing varieties which are identified by their texts. The instrumental folksongs commonly accompanied by drums in the region are used for dances.

* The analysis of Oromo folksongs of eastern Ethiopia, which is done for the first time from the perspectives of bioecocultural heritages, can be used as a starting point for future study. It is imperative to have a full-scale analysis from central, south, west and north parts of Oromia in order to get a holistic picture.

## Summary and conclusion

In summary farmers described the bioecocultural heritage of sorghum to their livelihood using folksongs. This indicated farmers' rich indigenous technical knowledge and technology in depicting qualitatively from land preparation to crop utilization. Farmers' knowledge should be capitalised. The following inferences can be implicated from these folksongs-based study of bioecocultural heritage:

1. Farmers have been growing sorghum for hundreds of years. The farmers' knowledge and sorghum have been co-evolving together. This has resulted in the prevalence of rich indigenous technical knowledge of the farmers. Any attempt to improve the crop needs to take into account the farmers knowledge and experience.

2. The crop's importance to the farmer is manifested by the multiple uses the crop renders, which was reflected in the folksongs. These are food, feed, fuelwood and construction materials. This is a paucity in other parts of the world. Despite the multiple uses, most of the attempts made by formal research were geared to food values only. Of course, this has resulted in poor adoption of improved varieties by the farmers [5,11]. Hence, formal sorghum breeding in Ethiopia needs to be reorientated into multipurposive sorghum variety development scheme.

3. Ethiopia is a centre of diversity for sorghum. This has resulted into biologically and ecologically diverse populations of sorghum ***in situ***. However, all the previous efforts made in the utilization of on-farm genetic resources have been non-participatory in its approach which resulted in limited utilization of farmer developed genetic resources. This led into breeding of varieties that has limited adoption by farmers. All future attempts in the improvement of the crop need to include farmers and their knowledge in the research-extension continuum.

4. The comparative strategic value of sorghum with other crops is shown by the farmers by folksongs. This actually corroborates the special importance of sorghum for farmers' livelihood in the region. Any activity in line with food security and improving agricultural productivity has to give *a priori *consideration to sorghum.

5. Over 95%, in the region are using varieties developed by themselves over years. The presence of *de facto *variation among the varieties is implied from simple description of farmers' varieties. The role of improved varieties in the region is very much limited. Any attempt to improve sorghum has to give emphasis on enhancement of farmers' varieties.

6. Farmers not only characterise sorghum at a folk species level but at varieties and sub-varieties level also. The formal characterisation and utilization of sorghum should consider the farmers' folksong based system for improved use of sorghum.

7. This is the first attempt made to use folksongs for describing bioecocultural heritage of sorghum diversity, production and utilization. The need for documentation of the oral knowledge in the bioecocultural heritage was found imperative for the following reasons: a) in the near future the oral traditions are most likely to be on the verge of being lost which necessitated the documentation that describes the bioecocultural heritage; b) the number of farmers is dwindling as land size and farming population is inversely related and most of the upcoming farming community are forced to go to work in the urban areas; c) farmers are mostly illiterate in order to document it in written form e) the young rural boys and girls are now going to the school and are becoming more of non-rural farming community f) In order to enhance sorghum productivity, the information on bioecocultural heritages are pillars for sustainable improvement and utilization of sorghum.

## Competing interests

The author declares that they have no competing interests.

## Supplementary Material

Additional file 1 **The cropping calendar of sorghum**. The data provided describes the different sorghum farming activities over the year. Click here for file

## References

[B1] FAO (2005). FAO statistical database.

[B2] Dogget H (1988). Sorghum.

[B3] CSA (Central Statistical Authority of Ethiopia) (2005). The preliminary result of area, production, and yield of temporary crops.

[B4] House LR (1992). A guide to sorghum breeding.

[B5] Mekbib F (2007). Infra-specific folk taxonomy of sorghum (*Sorghum bicolor *L. Moench) in the centre of diversity, Ethiopia: folk nomenclature, classification and criteria. J Ethnobiol Ethnomed.

[B6] Markoff J, Shapiro S, Weitman S (1975). Toward the integration of content analysis and general methodology," in Heise, David R. Sociological Methodology.

[B7] Pankhurst R (1961). An Introduction to Economic History of Ethiopia.

[B8] Huntigford GWB (1955). Galla of Ethiopia kingdom of Kaffa and Janjero.

[B9] Henze PB (2000). Layers of time A history of Ethiopia.

[B10] Westphal E (1975). Agricultural systems in Ethiopia.

[B11] Mekbib F (2006). Farmer and formal breeding of sorghum (*Sorghum bicolor *L. Moench) and the implication for integrated plant breeding. Euphytica.

[B12] Mekbib F, Farley C (2000). Participatory research for improved agro-ecosystems management in eastern Ethiopia, Alemaya wereda.

[B13] Ministry of Agriculture (MoA) (2000). Agro-ecological zones of Ethiopia.

[B14] Mekbib F, David S (1999). Informal bean seed systems in eastern Ethiopia.

[B15] Mekbib F (1997). Farmer participatory variety selection in common bean (*P. vulgaris L*.) genotypes in eastern Ethiopia. Experimental Agriculture.

[B16] Mekbib F (2002). Simultaneous selection for yield and stability. Journal of Agricultural Sciences Cambridge University.

[B17] Mekbib F (2003). Yield stability in common bean (*Phaseolus vulgaris *L.) genotypes. Euphytica.

[B18] Finnegen R (1970). Oral literature in Africa.

[B19] Leslau W (1946). Bibliography of the Semitic languages of Ethiopia.

[B20] Pound M (1968). Ethiopian music, an introduction.

[B21] Kebede A (1982). Roots of black music.

[B22] Elbourne R (1976). A mirror of man? Traditional music as a reflection of society. The Journal of American Folklore.

[B23] Lomax A (1968). Folksong style and culture-Washington. American Association for Advancement of Science.

[B24] Merriam AP, Bascom W, Herskovits M (1959). African music. continuity and change in African cultures.

[B25] Debalke T (1967). Ethiopian Music and Musical instruments. Ethiopian Herald.

[B26] Sarosi B (1970). Melodic pattern in the folk music of the Ethiopian peoples Proceedings of the third International Conference of Ethiopian studies.

[B27] Mondon-vidailhet FMC, Lavignac A, de la Laurencie L (1922). The Ethiopian Music Encyclopaedia of Dictionary of Music.

[B28] Kebede A (1971). The Music of Ethiopia: its development and cultural setting.

