# Supplementary material for: Folksong based appraisal of bioecocultural heritage of sorghum (Sorghum bicolor (L.) Moench): A new approach in ethnobiology
Source: J Ethnobiol Ethnomed. 2009 Jul 3;5:19. doi: 10.1186/1746-4269-5-19 (PMC2717052; doi:10.1186/1746-4269-5-19)

**Additional file 1. The cropping calendar of sorghum**

*(aata provided describes the different sorghum farming activities over the year)*

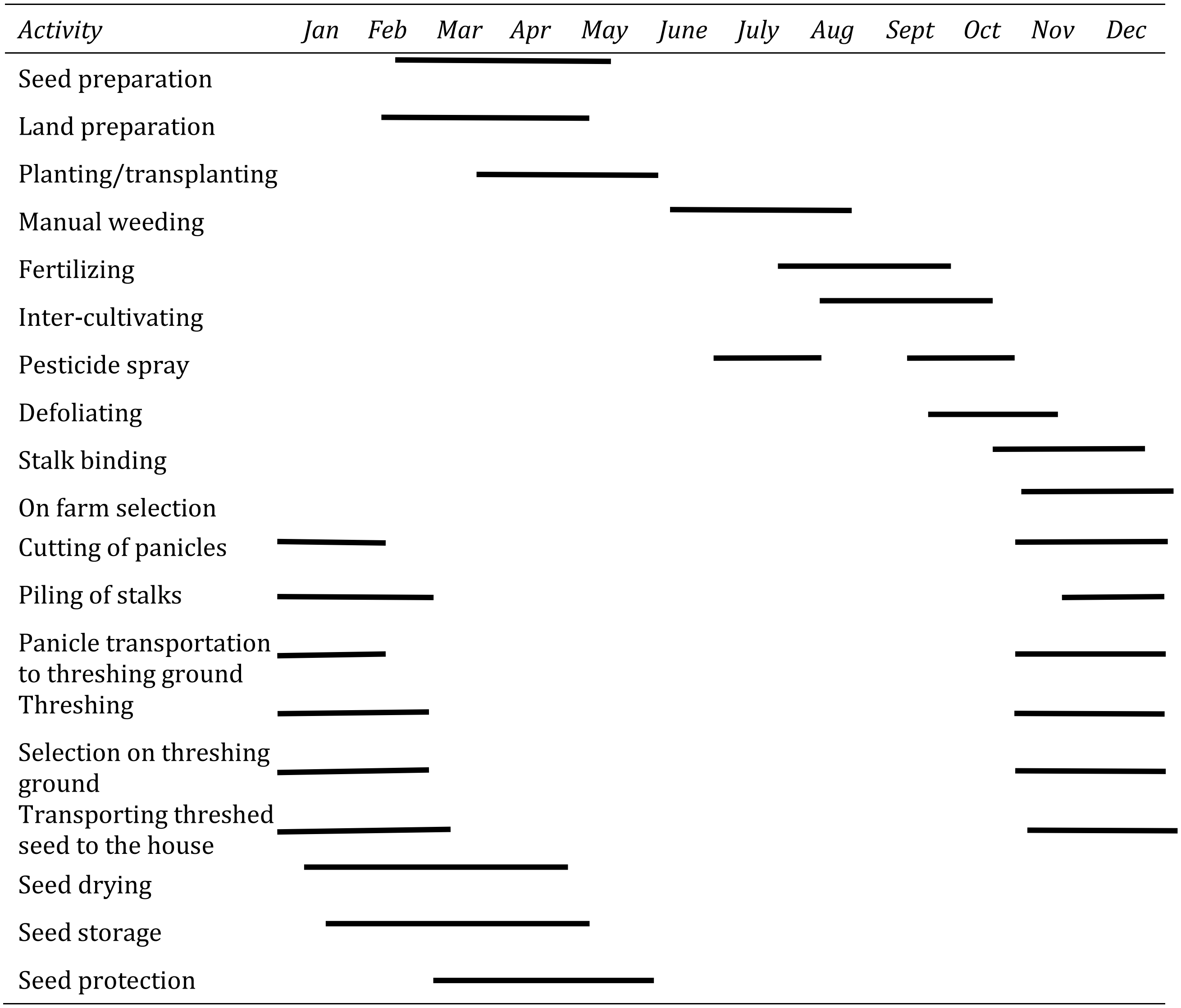

Supplement: Additional file 1 — The cropping calendar of sorghum. The data provided describes the different sorghum farming activities over the year. [file 1746-4269-5-19-S1.pdf]
